# Functional and Therapeutic Significance of Tumor-Associated Macrophages in Colorectal Cancer

**DOI:** 10.3389/fonc.2022.781233

**Published:** 2022-02-02

**Authors:** Yitong Li, Zhenmei Chen, Jiahao Han, Xiaochen Ma, Xin Zheng, Jinhong Chen

**Affiliations:** ^1^ Department of General Surgery, Huashan Hospital, Fudan University, Shanghai, China; ^2^ Cancer Metastasis Institute, Fudan University, Shanghai, China

**Keywords:** colorectal cancer, tumor-associated macrophages, mechanism, tumor microenvironment, treatment

## Abstract

The role of the tumor microenvironment (TME) in the progression of colorectal cancer (CRC) and its acquisition of resistance to treatment become the research hotspots. As an important component of TME, the tumor-associated macrophages (TAMs) regulate multiple critical oncogenic processes, namely, occurrence, proliferation, metastasis, and drug resistance in CRC. In this review, we have discussed the functional and therapeutic significance of TAMs in CRC. M1 macrophages act as the tumor suppressor while M2 macrophages promote CRC. The polarization of TAMs is mainly regulated by the pathways such as NFKB1 pathways, STAT3 pathways, WNT5A pathways, and PI3K pathways in CRC. Furthermore, the M2 polarization of TAMs is not only controllable but also reversible. Finally, we provide insights into the TAMs-targeted therapeutic strategies.

## Introduction

CRC is the third common cancer in males and second in females, causing 600,000 deaths per year (1). Surgery, chemotherapy, radiotherapy, and targeted therapy are currently established as commonly used options for CRC patients (2–4). With advances in therapeutic strategies, the prognosis of CRC patients has been tremendously improved (5). Patients with stage IIIC colon cancer have a 5-year survival rate of 53%, while 58% for stage IIIC rectal cancer (6). However, drug resistance of CRC is common that prevents treatment from achieving the expected outcome (5, 7). The resistance mechanisms have been observed in CRC cells, namely, promoting degradation of drugs, inhibiting apoptosis, affecting drug delivery, interfering DNA damage and repair, regulating epigenetic factors, and inducing cell cycle arrest (4, 8–10). The treatment effect is not very satisfactory due to resistance. Patients with BRAF V600E-mutated metastatic colorectal cancer only have a median overall survival of 4 to 6 months due to multidrug resistance (11). Therefore, effective strategies are urgently needed to improve patient prognosis.

At present, TME plays an essential role in CRC and gradually gains prominence as a new therapeutic target for the reversal of resistance (12, 13). Advances in the understanding of the TME have contributed to the exploration of treatments for advanced CRC (14). To date, pembrolizumab and nivolumab, two anti-programmed death protein 1 (PDCD1) inhibitors, were approved by the FDA for treating mismatch-repair-deficient (dMMR) and microsatellite instability-high (MSI-H) metastatic colorectal cancer (mCRC) (15). Although clinical data have revealed that immunotherapy can double progression-free survival (PFS), the treatment can only be applied in patients with MSI-H-dMMR CRC, accounting for 15% of all CRC cases (16, 17). The limitation has prompted the search for novel therapeutic targets to improve clinical applications and the effects of immunotherapy.

TME is a complex network comprising cells (T cells, B cells, TAMs, myeloid-derived suppressor cells, cancer-associated fibroblasts, etc.) and non-cellular components (cytokines, proteins, oxygen, etc.) (18–22). As the main component of TME, TAMs can interact with tumor cells by secreting cytokines, participating in CRC processes (23–26). For example, TAMs secret CXCL8 triggering EGFR signaling of tumor cells which is the leading cause of drug resistance in refractory CRC with KRAS or BRAF V600E mutation (27). The expression of immune checkpoints such as PDCD1 in TAMs contributes to immunosuppressed microenvironment in CRC (25, 28–32). With high plasticity and heterogeneity, functions of TAMs in CRC are not limited to immunosuppression, and the M1 phenotype in TAM acts as an immunostimulator (33–35).The phenotypic plasticity in TAMs provides new insights for treatment. A lot of evidence has also revealed that phenotypes and infiltration of TAMs were related to the prognosis of CRC patients (36–39). Consequently, TAMs-targeted therapy is beneficial to improve therapy effectiveness and patient prognosis. The article aims to discuss the multiple roles of TAMs in CRC and summarize emerging treatments based on TAMs.

## TAMs Interact With Components of TME

The development of novel anti-CRC treatment has been challenged by the complex tumor environment (40). Many studies also demonstrate that increased pro-tumor cells (such as T-reg cells) and decreased anti-tumor cells (such as CD8 T cells and NK cells) are responsible for tumor escaping immune surveillance (41, 42). Hence, understanding the tumor microenvironment is essential to develop efficient treatment strategies. The TME consist of cancer cells, tumor-associated neutrophils (TANs), myeloid-derived suppressor cells (MDSCs), cancer-associated fibroblasts (CAFs), tumor infiltrating lymphocytes (TILs), dendritic cells and all kinds of cytokines, and has been gradually confirmed to improve the malignant potential of tumors (43). TAMs, acting as an important component of TME, can not only directly act on tumor cells but also regulate other constituents of the TME, thus affecting tumor progression (44).

TANs have functional similarities to TAMs. Both cells above exert a dual effect on CRC development with several isoforms, but the interactions between TAMs and TANs in CRC need to be further investigated (45, 46). MDSCs possess immuno-suppressive properties (47). In CRC, TAMs could shape the inflammatory microenvironment by secreting cytokines to promote MDSC production and the recruitment of MDSCs contributes to the generation of TAMs (47, 48). This activation loop between TAMs and MDSCs help tumor expansion and immune evasion. Furthermore, TAMs induce matrix deposition and collagen fibrillogenesis by increasing the expression of collagens XIV and I in CAFs, forming tissue barriers for CRC invasion and metastasis (49). Similar to MDSC, CAFs also act on TAMs. Studies have shown that immunosuppressive factors secreted by CAFs induce polarization of M2-like TAMs (50). In terms of CRC prognosis, both TAMs and TILs have the potential to predict tumor recurrence and survival in CRC patients and the high density of TAMs and TILs indicates a longer survival (51). But the correlation between TAMs and TILs, just like that between TAMs and dendritic cells, still need further exploration (52). One of inflammation hallmarks of TAMs is the cytokine secretion (53). For example, the IL6 secreted by TAMs could promote CRC self-renewal and metastasis (54). TAMs can also expel proteins and enzymes including DOCK family through vesicle transport to mediate the activation of lymphocytes (55). The polarization procedure of TAMs requires the participation of cytokines including IL4, IL10 and so on (56, 57).

As we can see, TAMs interact with multiple constituents in TME to participate in the regulation of CRC. However, the current studies about the interaction between TAMs and immune-related factors are imperfect, which limits our ability to develop specific therapeutic treatments. It is undeniable that TAM plays an important role in CRC and further exploration of its specific role in the various processes of CRC should be conducted in order to find breakthrough points in interventions.

## The functions of TAMs in CRC

**Figure 1:**
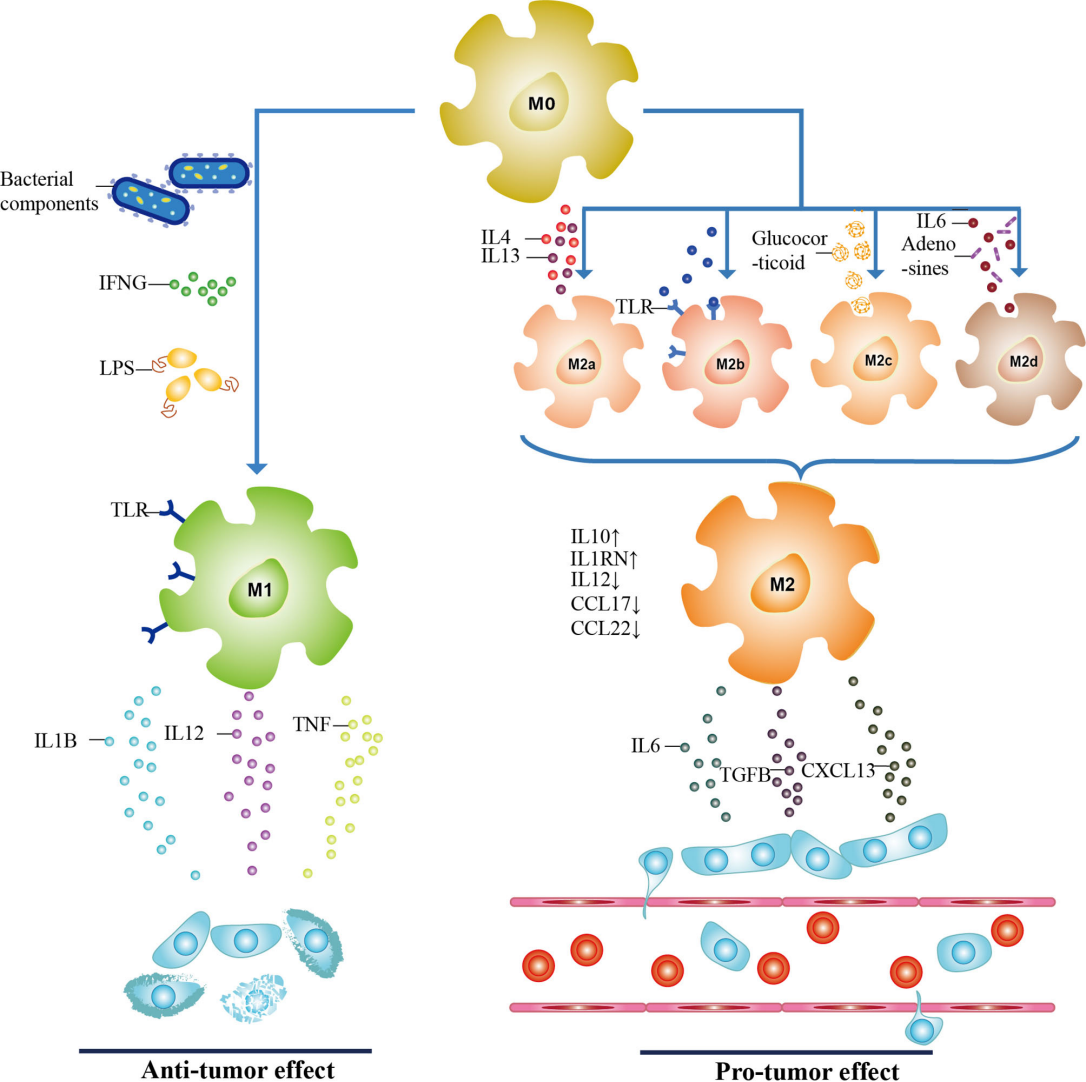
The polarization of TAMs. TAMs have three subtypes, including undifferentiated macrophages (M0 phenotype), pro-inflammatory phenotype (M1 phenotype), and anti-inflammatory phenotype (M2 phenotype). When activated by bacterial components, interferon-γ (IFNG), lipopolysaccharide (LPS), and Toll-like receptor (TLR), M0 macrophages are polarized toward M1 through classical activation. With a high expression of IL6 and CXCL8, and lower levels of CCL22, CCL17, and IL12, M2 macrophages are generated through alternative activation of M0 macrophages. Depending on stimulators, they can be further classified into M2a, M2b, M2c, and M2d subtypes. M1 and M2 phenotypes secret cytokines to participate in tumor progression, and the role of both in CRC is antagonistic to each other. It is confirmed that M1 macrophages act as the tumor suppressor while M2 phenotype promotes CRC.

Initially, TAMs exist in the form of undifferentiated macrophages M0, and under some conditions, M0 could be polarized into M1 (generated by classical activation) or M2 phenotype (generated by alternative activation) (**Figure 1**) (58, 59). Stimulators of classical pathway, namely, bacterial components, interferon-γ (IFNG), lipopolysaccharide (LPS), and Toll-like receptor (TLR), polarize M0 into M1 phenotype, which exerts cancer inhibitory effect by releasing pro-inflammatory cytokines (such as IL1B and IL12) and cytotoxic substances (such as reactive oxygen species and TNF) (60–62). While M2 macrophages display tumor-promoting activity and can be further classified into four different subtypes, M2a (induced by IL4, IL13), M2b (induced by TLR), M2c (induced by glucocorticoid), and M2d (induced by IL6 and adenosines) (63). *In vivo*, with lower CCL22, CCL17, and IL12 expression, M2 cells secrete a series of molecules such as IL6 and CXCL8 to exert immunosuppressive functions (60, 64). Further insight into the functions of TAMs in CRC is the basis for effective therapeutic targeting.

### CRC Occurrence

A prospective cohort study showed the amount of TAMs could change the effect of smoking on CRC occurrence (65). However the mechanisms of correlation between TAMs and CRC occurrence remain unclear. Cancer Stem Cell (CSC) is a kind of cancer cell phenotype having abilities of self-renewal, clonal tumor initiation, and rapid proliferation, taking part in tumorigenesis as the primary cell group (66). Therefore, in this part, we focus on the interaction between TAMs and CSC. In cancer cells, high expression of T-cell immunoglobulin domain and mucin domain 4 (TIMD4) and SIX1 protein indicates upregulated CSC-like properties. These cells would recruit TAMs and promote M2 polarization, leading to tumor interstitial remodeling (67, 68). In addition to proteins, RNAs regulate the functions of CSC. CSC could downregulate several microRNAs through a circRNA–microRNA–mRNA axis, thus assist the aggregation of downstream molecules, including SKIL, SMAD2, WNT5A, and so on (69).

Meanwhile, in clinical samples, it was found that overexpression of WNT5A could polarize TAMs into the M2 phenotype and regulate IL10 secretion (57). Nevertheless, the reason for the upregulation of WNT5A remains unclear. As in mammalian cells, microRNAs would be packed into exosomes to discharge and function extracellularly (70). There might be a possibility that CSC excretes microRNAs to connect with TAMs. Simultaneous, CSC is regulated by TAMs, and recent *in vivo* studies suggest the proportion of CD68^+^ TAMs at the tumor invasive front (TF) is correlated with the level of stem cell marker CD44v6 (71, 72). After infiltrating in the cancer area, M2 subtypes increase the ratio of CD44^+^/CD166^+^ tumor cells and the expression of acetaldehyde dehydrogenase 1 (ALDH1), which is the marker of CSC (57, 67, 73).

### CRC Proliferation

Many components of the immune microenvironment are implicated in proliferation, which is a primary hallmark of tumor (74). At present, the role of TAM in CRC proliferation has been widely reported. Through secreting transforming growth factor β1 (TGFB1), TAMs have abilities to upregulate vascular endothelial growth factor (VEGF) and interleukin-6 (IL6), and the latter binds with IL6 receptor on the tumor cell surface to promote CRC proliferation *via* activating STAT3 (75, 76). The extent of STAT3 activation is affected by diet, and dieting is considered a potential therapy that promotes anti-tumor immunity (77). Under fasting conditions, M2 polarization was inhibited, resulting in limited proliferation and increased apoptosis of CT26 colon cancer cells (78). A current study provided an important observation that serum starvation caused differentiation markers of T-reg, TGFB1, and FOXP3 rising (79). This evidence leaves us some questions that if TAMs could play a role in T-reg by modulation of TGFB1, fasting combined TGFB1 neutralizing antibody might show more potency against CRC proliferation. Based on the above research, eliminating M2 TAMs is regarded as an effective anti-cancer therapy. Yet, in eliminating macrophages of Ccr2^−/−^ mice, TAMs can still proliferate and release cytokines to promote the growth of CRC (80). So, in addition to the activation of the M0 alternative pathway, there are other ways to generate M2 macrophages. Meanwhile, CRC could escape immune supervision through this bypass, and figuring out associated specific mechanisms would bring new understanding to clinical therapy.

### CRC Metastasis

During the malignant transformation, cancer cells undergo function acquisition and alteration due to genetic mutation (81). Among all the functions, the capacity of invasion is an essential criterion for judging malignant diseases (82). Many studies have observed that TAMs accelerate tumor invasion mainly by regulating the epithelial–mesenchymal transition (EMT) process during which tumor cells gradually discard epithelial characteristics and obtain mesenchymal phenotypes, generating circulating tumor cells and tumor stem cells (83, 84). After a positive correlation between TAMs and the EMT marker snail was observed, TAMs were confirmed to release transforming growth factor β (TGFB) to activate the TGFB/Smad2,3-4/Snail signaling pathway, and restraining the pathway with TGFB receptor inhibitor might reverse metastasis (84). Apart from TGFB, TAMs secret other cytokines, namely, IL4 and IL6, and the former indicates the formation of M2 phenotype, while the latter activates the STAT3 signaling pathway to trigger EMT in CRC (56, 75). EMT is an inter-regulating course, and there is an urgent need to lock the TAM/EMT axis. In recent years, intestinal microbiota has attracted much attention in immunomodulation (85, 86). The microbiota dysfunction can induce TAMs secreting IL6 and TNF to launch EMT of HT29 colorectal adenocarcinoma cells (87). Maybe intestinal microbiota is an excellent target to attenuate the M2 phenotype. In-depth analysis of M2 macrophage-derived exosomes (MDE) shows high expression of miR-155-5p, while the microRNA also participates in metabolism-related gene transcription of intestinal microflora (88, 89). The findings provide the basis of intestinal microflora regulation served as one target of inhibiting CRC metastasis.

### Prognosis in CRC

The degree of macrophage infiltration can indirectly reflect the prognosis of patients (90). The risk of recurrence and cancer-associated mortality was doubled in patients with high M2 proportion in lesion compared with low M2 infiltration, and the high invasion of M1 macrophages in tumor matrix predicted a good prognosis and extended survival period (91). To further substantiate this, a subset of 168 patients with stage II/III colorectal cancer received follow-up interviews after operation. The results demonstrated that the patients with a high density of CD163^+^ TAMs have worse overall survival (OS) and progression-free survival (PFS) (92). For CRC patients, early detection and diagnosis are of great significance for a good outcome, and carcinoembryonic antigen (CEA), the most used biomarker for noninvasively detection, is less effective due to poor sensitivity (93). As same as CEA, TAMs are also distributed in the blood and barely influenced by surgery status (91, 92, 94). Prognostic information can be acquired by analyzing the proportion and polarization state of TAM, such as the ratio of CD206^+^/CD68^+^ TAM (95). A study consisting of 931 colorectal cancer patients showed the high M1/M2 ratio was suggestive of lower mortality in colorectal cancer (96). In a word, the infiltration density and polarization state of TAMs influence the prognosis of patients, yet the potential for reflecting prognosis needs further more clinical research (39).

## TAMs and Aberrant Signaling Pathways

**Figure 2:**
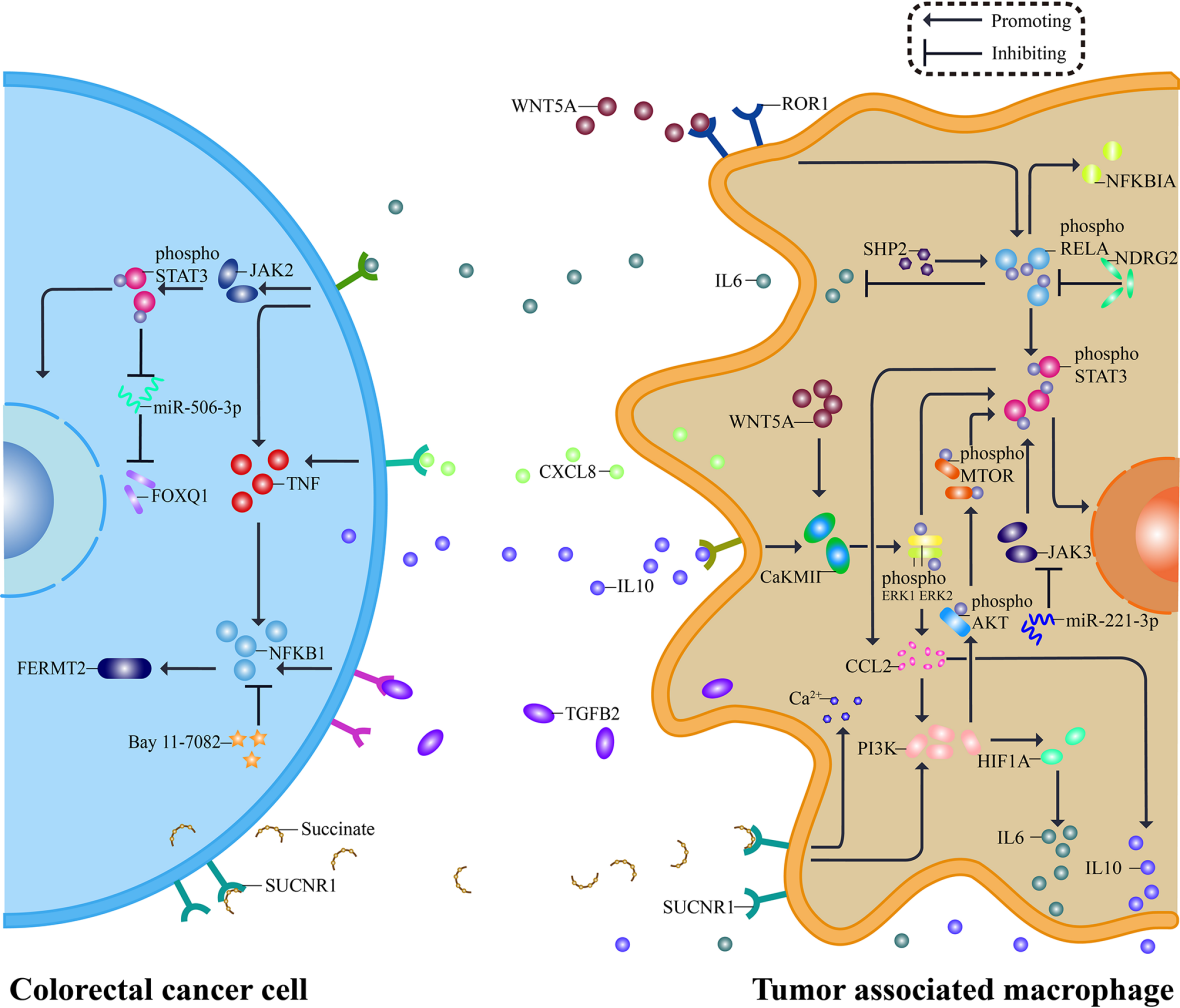
Pathways between TAMs and CRC. The interaction between CRC and TAMs relies on a complex molecular network. Tumor cells release molecules such as IL10 and Succinate, activating STAT3 pathway and PI3K pathway in macrophages. Consequently, pro-tumor cytokines, including IL6, CXCL8, and TGFB2, are secreted out of TAMs and targeted to their receptor on the surface of CRC cells. Specific inhibitors aiming at essential pathway points effectively reverse the pro-tumor process, typically miR-221-3P inhibiting STAT3 pathway by silencing JAK3. Besides, WNT5A and NFKB1 contribute to the network, and each pathway is not independent but interconnected. The secreted pro-tumor cytokines cause the response of JAK2, TNF, and NFKB1, serving CRC. A similar network can be seen both in TAMs and CRC, and suppressors inside it may have a dual function.

Colorectal cancer is a highly heterogeneous disease due to the disorder of cell signal regulation mechanism (97). Abnormal activation of pathways is involved in the CRC cell-TAM regulatory loop, which contributes to tumor biology (98, 99). Consequently, the pathway is like a bridge to connect tumor and TAMs, and TAMs have been proved to be involved in many pathways important to CRC,namely, NFKB1 pathway, STAT3 pathway, WNT5A pathway, and PI3K pathway (**Figure 2**) (75, 98, 100, 101). While less evidence indicates tumor associated macrophages interact with Hedgehog pathway and Notch pathway, both of which are concerned with regeneration and renewal of the epithelium, this may be a pointcut for better understanding the role of TAMs in CRC (102, 103). This part will summarize the interaction between TAMs and four pathways frequently reactivating in colorectal cancer.

### NFKB1 Signaling Pathway

Src homology 2 domain-containing tyrosine phosphatase 2 (SHP2) has a tumor inhibitory effect in CRC. When SHP2 is deficient in TAMs, the phosphorylation of RELA protein is declined. It facilitates the polarization of TAMs to M2 phenotype, which can release IL6 and CXCL8 to increase the expression of TNF in CRC cells, leading to the activation of NFKB1 pathway and then promotes tumor angiogenesis and metastasis (26, 104). This procedure emphasizes the importance of epigenetics in TAMs. And enhancing the phosphorylation levels of RELA by knocking out N-myc downstream-regulated gene 2 (NDRG2) leads to IκBα upregulating. As a result, the monocyte polarization is induced toward M1-like macrophages, playing an inhibitory role in tumor (105). Additionally, M2 macrophages can secrete TGFB2 and activate NFKB1 pathway to regulate FERMT2, forming the TGFB2/NFKB1/FERMT2 axis to promote CRC cells invasion, and NFKB1 inhibitor Bay 11-7082 can reverse functions mentioned above (106, 107). Though there are no findings of associated clinical trials by far, all kinds of inhibitors have brought hopes for the treatment.

### STAT3 Signaling Pathway

STAT3 is a component of the IL6 activated acute phase response factor (APRF) complex, which can be activated by various cytokines or growth factors (108). As we mentioned before, TAM can release cytokines, especially IL6, which could bind specific receptors on CRC cell surface, activating JAK2/STAT3 signaling pathway, downregulating tumor suppressor miR-506-3p, and relieve the inhibition of the latter on FOXQ1 that results in enhanced invasion and metastasis of CRC (25). Inhibition of this signaling pathway can reverse CRC metastasis caused by nicotinic acetylcholine receptor α7 (α7nAChR) knockout (107). Like IL6, IL10 secreted by CRC could induce TAM polarization to M2 macrophages through CaKMII/ERK/STAT3 pathway and promote cancer cell itself (57). On the contrary, with the effect of miR-221-3p for silencing JAK3/STAT3 activation, M2 TAMs begin exhibiting characteristics of M1 (109). The STAT3 pathway is not a one-way path, but a loop between CRC and TAMs, and the circle could be disrupted if the pathway was interrupted, which needs a lot more research exploring the association between TAMs and members in this loop.

### WNT5A Signaling Pathway

WNT5A is a secreted glycoprotein belonging to the WNT family, and related signaling pathways mainly regulate cell proliferation, angiogenesis, and so on (110). As for CRC, WNT5A is expressed primarily in tumor matrix, especially in TAMs, but its specific biological function and related mechanism are not fully understood, which needs further exploration (57). WNT5A can form a cascade with CaKMII and ERK1. When the pathway is activated, TAM will be polarized to M2 phenotype and release IL10, aiming to promote the progression of tumor (57, 100). For the tight association of WNT5 with chemokine ligands, researchers subsequently focused on another molecule CCL2, and it was interesting that when WNT5A was used to treat undifferentiated macrophages, CCL2 mRNA was upregulated most significantly among all cytokines (111, 112). After silencing WNT5A expression, the level of ERK activation and expression of CCL2 declines, consequently inhibiting M2 polarization (111). As we all know, non-single but multiple pathways involve in CRC. For instance, the WNT5A pathway could link to the NFKB1 pathway under the action of ROR1, then sensitizing STAT3 (113). Therefore, the WNT5A signaling pathway plays an important role in CRC malignant biological behavior and macrophage infiltration, yet we still know little about how different pathways connect.

### PI3K Signaling Pathway

Phosphatidylinositol-3-kinase (PI3K) signaling pathway is intracellular, which can be activated by lots of cytokines, affecting TAM recruitment and polarization (114). Tumor cells can release succinate acting on macrophages and promote their transformation through the PI3K–hypoxia–inducible factor 1α (HIF1A) axis (22). MiR-934 is one of the activating factors released by exosomes, downregulating the expression of PTEN in TAM, consequently activating the PI3K/AKT1 signaling pathway to induce the M2 polarization (115). In addition to microRNA, CRC can also release type Iγ phosphatidylinositol phosphate kinase (PIPKIG) to trigger PI3K/AKT1/MTOR signaling pathway, promoting TAM recruitment in tumor area through reinforcement of CCL2 transcription, thus providing an appropriate environment for CRC development (116). M2 macrophages also release CXCL13 to trigger CXCL13/CXCR5/NFKB1 signaling to induce CRC liver metastasis (115).

## TAM Associated Treatment

**Figure 3:**
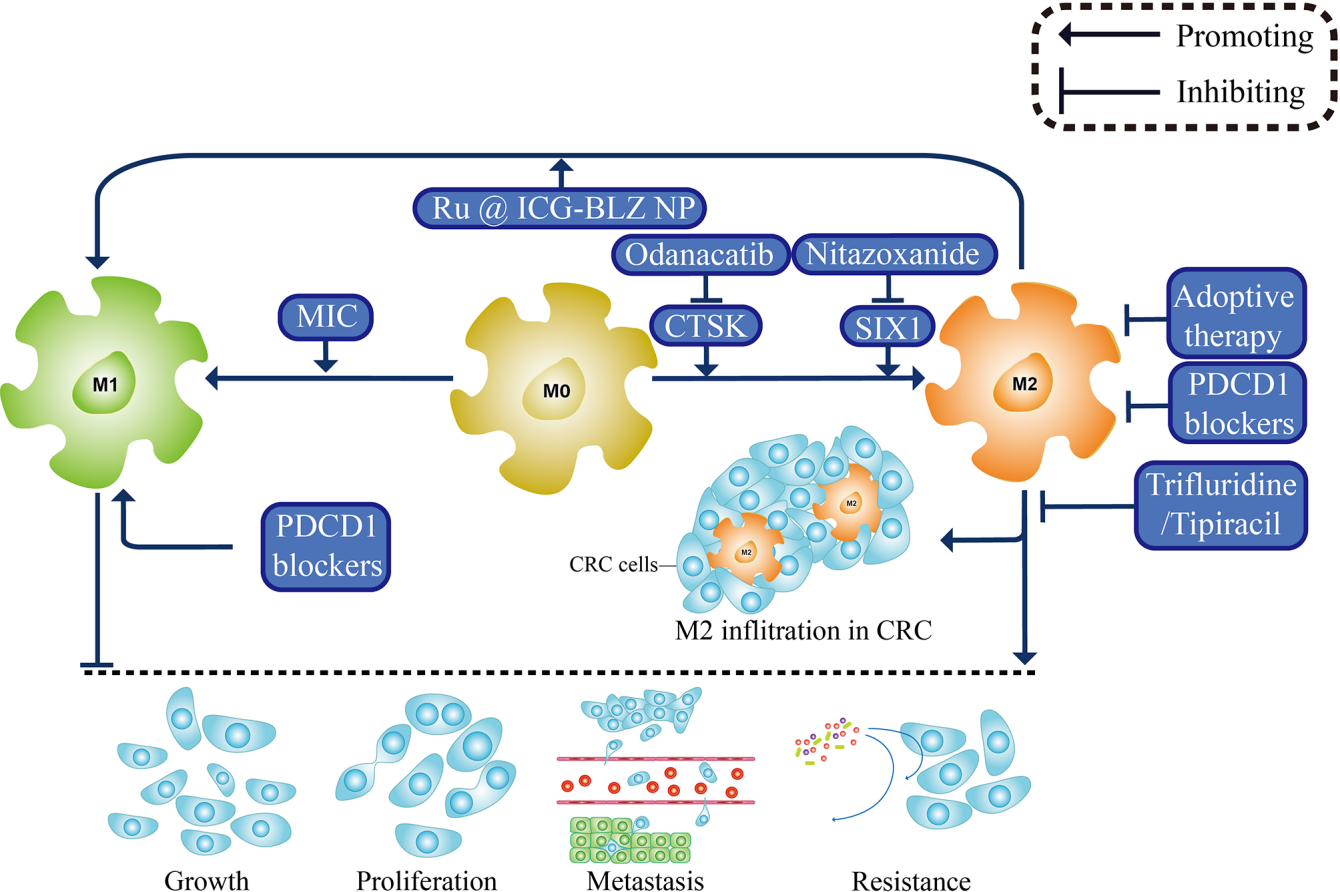
Treatments based on TAMs. M0 macrophages polarized towards M1 or M2 phenotypes to participate in growth, proliferation, metastasis, and drug resistance of CRC. Therefore many treatments are designed based on the polarization of TAMs. Odanacatib and Nitazoxanide, targeting CTSK and SIX1 respectively, both inhibit the alternative activation of undifferentiated macrophages, and MIC triggers the polarization of M1 macrophages. Meanwhile, nanoscale drug Ru @ ICG-BLZ NP can directly induce the conversion of M2 phenotype to M1 phenotype, showing a new method for clinical transformation. The role of cholesterol metabolism in CRC is gradually being valued, and a new anti-metabolism drug Trifluridine/Tipiracil was shown to weaken infiltration of M2 macrophages. Other therapy such as PDCD1 blockers and adoptive cell therapy could also act on TAMs and showing anti-tumor impact.

Systematic chemotherapy is the standard treatment for patients with advanced CRC, which can effectively prolong the overall survival time. Whereas the survival rate of patients with stage IV CRC is only 10%, drug resistance was one of the causes of this phenomenon (117). With deepening research, TME immune components are shown to participate in resistance, which is demonstrated by great strides in immunotherapy (118, 119). Primarily programmed by TME, TAMs have a significant impact on the treatment efficiency of CRC (54, 120). It was found that the conditional medium from M1 could improve the oxaliplatin sensitivity in tumor-bearing mice and that from M2 reduces the sensitivity of CRC to 5-fluorouracil (5-FU), suppressing caspase-mediated apoptosis to protect tumor cells from chemotherapy (121, 122), whereas reducing TAM proportion or inducing M1 directional polarization can effectively increase the survival time of patients and improve the prognosis (123, 124). Though M1 and M2 macrophages antagonize each other, interference of TAM gene expression by exogenous application of drugs can reduce the proportion of M2 TAMs in tumor infiltration regions and promote the transformation from M2 to M1 phenotype exerting tumor inhibition (**Figure 3**) (58, 105, 125). Additionally, the drug tolerance initiated from TAMs is concerned with effluxion. Given the difficulties in finding novel therapeutic targets, efflux inhibitors specifically targeted M2 subtype might work miracle.

### TAM as a Target for Immunotherapy

Immunotherapy, namely, immune checkpoint inhibitors, adoptive cell therapy, and tumor vaccines, has been popularized in CRC treatment (126). Programmed cell death protein 1 (PDCD1) is an immune checkpoint receptor. It has been reported that in CRC patients with high microsatellite instability, PDCD1 is highly expressed in M2 macrophages at the invasive region of CRC (127). The high PDCD1 expression of M1 phenotype, resulting in the degeneration of TAM phagocytosis, is positively correlated with disease state of CRC (128). PDCD1 blockers can enhance phagocytic ability of macrophages and prolong survival time of patients, confirming that PDCD1 therapy can directly aim at TAM. Besides, patients with more M2 macrophages infiltration in lesion areas have the potential to acquire better efficacy. As for adoptive cell therapy, the anti-tumor efficiency of the combination of tumor-directed anti-mesothelin CAR-T cells and M2 inhibitors has been verified, and TAM-associated adoptive cell therapy based on its specific markers, such as CD40, are being investigated (129). In cell models that stably express ovalbumin (OVA) peptide, the OVA vaccine could reduce the density of TAMs in CRC tissue, thus limiting tumor growth, and supplemental application of VEGFC/VEGFR3 neutralizing antibody could further inhibit the chemotaxis of M2 macrophages into CRC area, preventing CRC from escaping immune monitoring (130).

### Directional Regulation of TAM Polarization

The long non-coding RNAs (lncRNAs) serve as both non-invasive biomarkers and targeted molecules in CRC (131). For instance, lncRNA RPPH1 secreted by CRC cells mediates M2 polarization, promoting tumor metastasis, but is lacking independent means of intervention (132). Intestinal microflora, an important regulator of intestinal microenvironment, has been shown to modulate lncRNA gene expression in various tissues (133). Additionally, microflora can also induce M0 to M2 phenotype polarization by secreting cathepsin K (CTSK), which binds to toll-like receptor 4 (TLR4), activating MTOR pathway (134). CTSK-specific inhibitor Odanacatib is administrated to curb the related pro-tumor effects, improving the prognosis of CRC patients (134). In addition to the regular medications, researchers nowadays exploited nanoparticles with inflammatory molecule releasing ability, Ru @ ICG-BLZ NP, which has high CRC specificity and low toxicity (125). It can release CSF-1R kinase inhibitor BLZ945 and repolarized TAM to M1 macrophages to show an anti-tumor effect which provides a new idea for clinical transformation of nanodrugs (125).

### Inhibition of TAM Recruitment or Infiltration

Homologous protein SIX1 is widely expressed in all kinds of cancers, and its overexpression results in upregulation of macrophage-specific colony stimulating factor, thus recruiting pro-tumor TAM in the CRC region (68). Its inhibitor, Nitazoxanide, is expected to silencing SIX1 by suppressing WNT/CTNNB1 pathway (135, 136). Metabolism disturbance could partly explain the mechanism for TAMs inducing drug resistance. Trifluridine/Tipiracil is a new anti-metabolism drug, and its combination with oxaliplatin can effectively exhaust M2 macrophages, thereby causing cytotoxic CD8^+^ T cell infiltration to compel tumor cell lysis (137). Through years of treatment, clinicians observed a strange phenomenon that patients with colitis have a specific resistance to CRC, but the mechanism is unknown. One of the benign disease characteristics is chronic inflammation that persists for a long time, and many inflammatory factors are involved in this process. Macrophage inhibitory cytokine (MIC) is one of them, which can recruit M0 macrophages and T cells to lesion sites and exert tumor immune regulation (138). Subsequently, more clinical trials are required to explore the anti-CRC effect of MIF biological agents. The combination between it and conventional therapy may provide more options for clinical CRC treatment.

## Discussion

A growing understanding of the functions and regulatory mechanisms of TAMs will enable us to explore their future clinical applications in CRC. In this review, we described different subtypes of TAMs and their associated mechanisms in colorectal cancer. Based on the above, TAMs-related treatment for CRC was summarized with potential for further research. Many factors are associated with the poor prognostic of CRC, especially the low early diagnostic rate and poor pharmacological reaction. Therefore, it is of great significance to explore the relevant mechanisms and early diagnostic methods beneficial to clinical prevention and treatment of CRC. TAM is an important component of the TME that mediates CRC proliferation, metastases, drug resistance, and so on. Once stimulated by tumor-associated factors, TAMs migrate to the tumor area at first, occurring polarization to M1 or M2 phenotype, and exerts tumor inhibition or promotion effects, respectively. Nevertheless, whether TAM already exists or transferred from the circulatory system in the CRC tumor area is still doubtful. Additionally, the function of TAM is mostly indirectly proved, and clinical research focusing on TAM is still lacking.

TAMs secrete cytokines (IL6, IL10, etc.) and exosomes (miR-21-5p, miR-155-5p, etc.) through NFKB1 signaling pathway, STAT3 signaling pathway, and other pathways to act on tumor cells and immune cells, regulating the process of CRC. Moreover, TAMs secrete many immunomodulatory proteins in the form of vesicles to consolidating the tumor-promoting roles of stromal cells. Apart from acting on tumor cells, TAMs could regulate other immune cells of TME, thus participating in the regulation of immune microenvironment. That phenomenon may be the cause of treatment failure. Drug resistance is one of the most troublesome problems during the treatment of CRC, and TAM polarization is closely related to the resistance. Especially, M2 phenotype can secrete various factors such as CCL2 to weaken the sensitivity of CRC to oxaliplatin and other drugs, which suggest that TAM is expected to become a tumor therapeutic target. The induction of directional differentiation *in vitro* is helpful to understand the capacity of different TAM subtypes further. However, the progress of TAM study is slow due to the difficulty of establishing associated models, and there is a long way to address this issue.

Both immune checkpoint inhibitor PDCD1 and tumor vaccine are capable of inhibiting M2 formation or promoting its depletion. PDCD1 takes TAM as a direct target to inhibit tumor progression. The development of nanoscale drug load particles, such as Ru @ ICG-BLZ NP, provides a new method for the clinical transformation of nanodrugs. Since TAMs could release cytokines, neutralizing antibodies against M2 macrophages associated factors also show high anti-tumor value. Limagne et al. (137) found that TAM could also guide direction of T cell infiltration and migration. Therefore, in addition to regulating macrophage polarization states, TAM-targeted therapeutics can improve efficacies of immune checkpoint inhibitors. All of these have shown bright therapeutic prospects and great research potential. However, there are still some limitations. The specific functional mechanism of immunotherapy such as PDCD1 on TAM is still ambiguous. Although TAM is currently considered to be a biomarker, more clinical experiments are required to confirm its efficiency. Furthermore, reactive oxygen species (ROS) can actuate macrophage activation and function (139). Does ROS related mechanism attend TAM tumor regulation process? Whether metabolomics and biological rhythm also affect TAM function, these questions are worthy of further studies. The main biomarkers of TAM, such as CD68, transmembrane receptors, and secretory proteins, are expected to be therapeutic targets (120). Based on this, CRC-specific immunotherapy has been developed, which shows great application prospects and clinical significance.

We follow standardized nomenclature for gene products recommended by experts (140).

## Author Contributions

YL drafted the manuscript. ZC designed the figures and revised the manuscript. JC has done critical revision, and approved this version of the article. All authors listed have made a substantial, direct, and intellectual contribution to the work and approved it for publication.

## Funding

This work was supported by the National Natural Science and Technology Major Project of the thirteenth Five Year Plan (grant 2017ZX10203207) and the National Natural Science Foundation of China (grant 8200033791, 8207034966).

## Conflict of Interest

The authors declare that the research was conducted in the absence of any commercial or financial relationships that could be construed as a potential conflict of interest.

## Publisher’s Note

All claims expressed in this article are solely those of the authors and do not necessarily represent those of their affiliated organizations, or those of the publisher, the editors and the reviewers. Any product that may be evaluated in this article, or claim that may be made by its manufacturer, is not guaranteed or endorsed by the publisher.
